# Signs, Symptoms, and Side-Effects Presented by Different Types of COVID-19 Vaccines: A Prospective Cohort Study

**DOI:** 10.3390/life12122046

**Published:** 2022-12-07

**Authors:** Zahra Zare, Abdolghader Assarroudi, Mohammad Reza Armat, Mojtaba Laal Ahangar, Mahdie Estaji, Vahideh MoghaddamHosseini, Mostafa Dianatinasab

**Affiliations:** 1 Department of Midwifery, School of Nursing and Midwifery, Sabzevar University of Medical Sciences, Sabzevar 9617213112, Iran; 2 Iranian Research Center on Healthy Aging, Department of Medical-Surgical Nursing, School of Nursing and Midwifery, Sabzevar University of Medical Sciences, Sabzevar 9617213112, Iran; 3 Geriatric Care Research Center, Department of Medical-Surgical Nursing, School of Nursing and Midwifery, North Khorasan University of Medical Sciences, Bojnurd 7487794149, Iran; 4 Sheffeild Teaching Hospital, Sheffeild S5 7AU, UK; 5 Department of Midwifery, School of Nursing and Midwifery, Mashhad University of Medical Sciences, Mashhad 9137913199, Iran; 6 Iranian Research Center on Healthy Aging, Department of Midwifery, School of Nursing, University of Medical Sciences, Sabzevar 9617213112, Iran; 7 Department of Complex Genetics and Epidemiology, School of Nutrition and Translational Research in Metabolism, Maastricht University, 6228 GR Maastricht, The Netherlands

**Keywords:** Oxford AstraZeneca, Sputnik V, Sinopharm, Covaxin, COVIran Barekat, pandemic

## Abstract

The concern about post-COVID-19 vaccine complications still remains. In addition, the evidence on Sinopharm, Sputnik V, Covaxin, and, in particular, COVIran Barekat, as well as comparisons between them by dosage after post-vaccination, is scarce. This study aimed to investigate and compare the prevalence of self-reported post-vaccination signs and symptoms following the first and second doses of different types of COVID-19 vaccines. Research design and methods: This prospective cohort study was conducted on more than 1500 health professionals who had received at least one dose of any type of Sputnik V, Sinopharm, Oxford AstraZeneca, Covaxin, and COVIran Barekat vaccines in Iran. The survey questionnaire was sent to participants online, 28 days after receiving each dose of the vaccine. Results: About 73% of health professionals reported at least one post-vaccination sign or symptom, developing mostly within the first 12 h (69.9%) and lasting up to 12 h (59.0%). Pain and tenderness at the injection site, fever, and muscle pain were the most common post-vaccination signs and symptoms in all vaccines, which were significantly higher in the Oxford AstraZeneca vaccine (*p* < 0.001) for both the first and second doses. The incidence rate of all post-vaccination signs and symptoms was significantly higher in the first dose than in the second dose (*p* < 0.05). Conclusion: The Oxford AstraZeneca vaccine showed the highest incidence rate, onset, and lasting time of signs and symptoms in both doses; however, they were not life-threatening. The onset time of signs and symptoms was significantly higher for the COVIran Barekat and Oxford AstraZeneca vaccines in both the first and second doses.

## 1. Introduction

Since December 2019, the coronavirus disease 2019 (COVID-19), as a novel virus of the severe acute respiratory syndrome coronavirus 2 (SARS-CoV-2), has been spreading worldwide. It was known as a public health emergency of international concern on 30 January 2020 and a pandemic on 11 March 2020 by the World Health Organization (WHO) [1]. By 6 January 2022, WHO had received reports of 296,496,809 COVID-19 cases worldwide, including 5,462,631 deaths [1]. Coronaviruses (CoVs) belong to the “orthocoronavirinae” subfamily of the family Coronoviridae, which is classified into four main genera according to the differences in the genomic structure and phylogenetic relationships, including alphacoronavirus, betacoronavirus, gammacoronavirus, and deltacoronavirus. Alphacoronaviruses and betacoronaviruses exclusively infect mammals and cause respiratory illness and gastritis in humans and animals, respectively [2]. The CoV has the largest genome among RNA viruses, with a genome size ranging from 26 to 32 kilobases (kb), with a helical-shaped positive sense single-stranded nucleocapsid [3]. SARS-CoV-2 contains four structural proteins (spike, envelope, nucleocapsid, and membrane) and sixteen non-structural proteins (nsp1−16) [4]. To successfully address this devastating global challenge, WHO introduced vaccination against COVID-19 as a part of the overall strategy in the Strategic Preparedness and Response Plan 2021 (SPRP2021) [5]. Having access to vaccines against COVID-19 with high immunity, efficacy, and minimal side-effects is an urgent and vital solution for protecting lives around the world [6]. The striking efforts of scientists have resulted in the development of different platforms for COVID-19 vaccines. Currently, there are three categories of vaccines based on their various platforms, including messenger ribonucleic acid (mRNA) (Pfizer-BioNTech (BioNTech, Mainz, Germany) Moderna (Moderna, Cambridge, MA, USA), adenoviral vector vaccines (AstraZeneca–Oxford (Oxford University and British-Swedish company AstraZenec, UK), Johnson & Johnson (Janssen Vaccines, Netherlands & Janssen Pharmaceuticals, Belgium), and inactivated whole-virus vaccines (Sinofarm (Sinofarm, Beijing, China), Sinovac (Sinovac Biotech Ltd., Beijing, China), Bharat Biotech(Bharat Biotech, Telangana, India)) [7]. They have been urgently and conditionally approved by the WHO emergency use listing, the Food and Drugs Administration (FDA), and the European Medicines Agency (EMA) [8,9].

As of 5 January 2022, 58.6% of the world population has received at least one dose of the COVID-19 vaccine [8]. Despite many benefits of receiving vaccines to protect against COVID-19, serious concerns have been raised about their complications and consequences among people, particularly healthcare professionals [10]. Certain mild or moderate systemic side-effects such as headache, muscle pain, fatigue, fever, chills, and diarrhoea, as well as those related to the injection site, including swelling, pain, and redness, can happen within a few days but mostly in the first 48 h after receiving COVID-19 vaccines [11]. In some studies, adenoviral vector vaccines like Oxford AstraZeneca( (Oxford University and British-Swedish company AstraZenec, UK) showed the highest prevalence and severity of local and systemic side-effects compared with other COVID-19 vaccine platforms [12,13,14,15,16]. However, some rare adverse allergic reactions to the vaccines, such as anaphylaxis after receiving the first dose of the Pfizer-BioNTech vaccine and unusual blood clots combined with low levels of platelet levels after receipt of the AstraZeneca and Johnson & Johnson/Janssen vaccines have been documented [17,18]. According to reports to the Vaccine Adverse Event Reporting System (VAERS), there were almost 2 to 5 cases per million of anaphylaxis; 47 cases of thrombosis with thrombocytopenia syndrome (TTS) were associated with the Johnson & Johnson vaccine; and 7899 (0.0020%) deaths occurred by 20 September 2021 [19]. Although all available COVID-19 vaccines received emergency and conditional approval in a short time, their safety, quality, and standardization are still a significant concern. As a result of this, precise investigations and continuous monitoring of their possible side-effects across time are in demand to acquire further knowledge regarding the safety of these new vaccines [20]. 

From the beginning of the pandemic, health providers and medical staff have been at high risk of being infected with COVID-19 and, consequently, have likely been among the first eligible recipients of the COVID-19 vaccines. Therefore, the evaluation of post-COVID-19 vaccine symptoms in this target group who have a critical role in the management of this pandemic will provide important knowledge about the real-world safety of COVID-19 vaccines [21]. Moreover, all of the data that has been published so far has assessed the safety of one [22,23,24] or two [12,14] vaccines in use, and there is no comprehensive comparison between the type and severity of side-effects present after receiving the first and second doses of several vaccines.

### The COVID-19 Vaccination in Iran

In Iran, the first confirmed cases of COVID-19 were detected on 19 February 2020 in Qom, which is the seventh largest city in Iran, and then spread sharply around the country with devastating impacts on business and the economy [25]. An estimated 6,201,476 COVID-19 infections and 131,778 COVID-19-related deaths had been reported by 5 January 2022 [26]. Regarding COVID-19 vaccination, six vaccines have been approved so far for use in Iran, including Sputnik V, Sinopharm, AstraZeneca/Oxford, Bharat Biotech, COVIran Barekat, and Johnson & Johnson [27]. As of 26 December 2021, 59.50 million Iranians (70.00%) had received at least one dose of a COVID-19 vaccine [26]. The healthcare providers and medical staff at medical centres were the first recipients of COVID-19 vaccines, followed by the elderly as a high-risk group [28]. This study aimed to investigate and compare the prevalence of the presented signs and symptoms following the first and second doses of different kinds of COVID-19 vaccines among healthcare professionals. Moreover, we assessed the association of the presented post-COVID-19 vaccines signs and symptoms with socio-demographic characteristics, the presence of comorbidities, and a previous positive test for COVID-19.

## 2. Method

### 2.1. Study Population and Data Collection

This observational prospective cohort study was conducted on 1500 healthcare providers and medical staff working in health centres and hospitals in Sabzevar, Iran, from June to September 2021. Sabzevar is the second largest city in Khorasan-Razavi province, located in the northeast of Iran.

We first contacted eligible healthcare providers by phone and explained the study to them. In the case of their agreement (N = 1500), we then sent the survey questionnaire and written informed consent to them via email and other multiple media platforms, such as WhatsApp2.21.7.5 (210705001) and Telegram 7.4.2 (22271), 28 days after receiving each dose of vaccine and asked them to fill it out and return it to us three days later.

### 2.2. Ethics Approval and Consent to Participate

This study was approved by the Ethical Committee of the Sabzevar University of Medical Science, Sabzevar, Iran (IR.MEDSAB., REC.1400.038). All methods were performed in accordance with the relevant guidelines and regulations (Declaration of Helsinki). Written informed consent was asked from participants, including a statement that participation was voluntary. 

### 2.3. Inclusion and Exclusion Criteria

All healthcare providers and medical staff who had received at least one dose of any type of COVID-19 vaccine, namely, Sputnik V, Sinopharm, Oxford AstraZeneca, Covaxin, and COVIran Barekat (N = 1753), were eligible to enter the study. Those medical staff who did not receive any type of COVID-19 vaccine from June to September 2021 and those who did not complete the survey questionnaire over the following three days after receiving vaccine were excluded from the study.

### 2.4. Instrument 

We collected data through a survey questionnaires containing questions related to socio-demographic characteristics, comorbidities, previous COVID-19 positive tests and dates, the name of the received vaccine, post-vaccination local signs and symptoms (e.g., pain, swelling, redness, tenderness, itch, warmth, and swollen armpit glands), systemic signs and symptoms (e.g., headache, arthralgia, myalgia, chest pain, fatigue, fever, shiver and chills, diarrhoea, rash, leg swelling, palpitation, sleeplessness, nausea and vomiting, anaphylaxis), the onset and lasting time of signs and symptoms and their severity, and the method of their treatment (self-limiting, taking drugs, hospitalization). We constructed this survey questionnaire using previous reports and publications related to COVID-19 vaccine evaluations and clinical trials, and then it was reviewed and assessed by five expert healthcare professionals to confirm its readability and validity [14,22,23].

### 2.5. Statistical Analysis and Sample Size

In this study, the Cochrane sample size formula for proportion estimation (n = Z2.P [1-P]/E2) was used for sample size calculation, where n is the sample size, Z is the standard value for the reliability or significance level, P is the population proportion, and E is the level of precision or margin of error. As the proportion of post-vaccination signs and symptoms in the target population was unknown, we used a conservative value of 5 in the formula for this proportion (P). A confidence level of 95% (Z = 1.96), and a margin of error (E) of 0.0253 resulted in a sample size of 1500. In this study, the cluster sampling approach was used for sampling process. The descriptive analysis of data, including demographic characteristics, previous COVID-19 infections, comorbidity, and post-vaccination signs and symptoms by dose, was presented as frequency with percentages for categorical variables and mean with standard deviation (SD) for quantitative variables. To compare the general characteristics between different groups of vaccines, we used Kruskal–Wallis and chi-squared tests. In addition, we performed the pairwise comparison of the onset and lasting times of post-vaccination signs and symptoms between different groups of vaccines using the ANOVA test separately for the first and second doses. Moreover, chi-squared tests were employed to compare the incidence rate of post-vaccination signs and symptoms between different groups of vaccines by dose. The statistical significance level was set to 0.05 for all tests. We performed statistical analysis using SPSS version 22.0 (SPSS Inc. Chicago, IL, USA).

## 3. Results

### 3.1. Basic Characteristics

Of the 1753 people who were eligible, 1500 were accepted to participate in the survey. The mean age of participants was 36.37 (SD = 7.99), and more than half of them were female (56.6%). Nurses accounted for the largest proportion of the participants (33.1%). Nearly 60% of the healthcare providers had a history of COVID-19 positive tests confirmed via the semi-quantitative reverse transcription-polymerase chain reaction (sqRT-PCR) test and/or chest CT findings, and the vast majority of them (90.8%) did not mention any comorbidity (Table 1).

### 3.2. Characteristics of Post-COVID-19 Vaccination Signs and Symptoms

**Figure 1:**
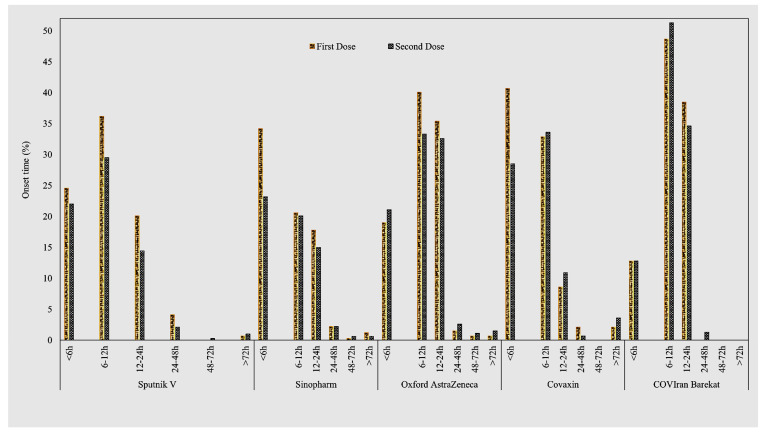
Frequency of Onset Time for Post-COVID-19 Vaccination Signs and Symptoms by Dose.

The COVID-19 vaccines Sputnik V (45.5%) and COVIran Barekat (0.05%) were the most and least frequently injected, respectively. The mean age of participants was highest for Sinopharm (37.5, SD = 8.9) and lowest for Sputnik V (35.5, SD = 8.0), and this difference was statistically significant (*p* < 0.01). In addition, the proportion of participants who had a positive test for COVID-19 was the highest in participants who received the Sputnik V (51.2%) and the lowest in those with COVIran Barekat (75.6%). This difference was significant (*p* < 0.001) (Table 2). Overall, approximately 73% of participants experienced at least one post-vaccine sign or symptom. The onset time of symptoms was mostly within the first 12 h (69.9%), and in the minority (4.7%), they happened within 72 h after vaccination (Figure 1).

With both the first and second doses of the COVIran Barekat and Oxford AstraZeneca vaccines, the onset time of symptoms was significantly higher compared to the other vaccines (Table 3).

**Figure 2:**
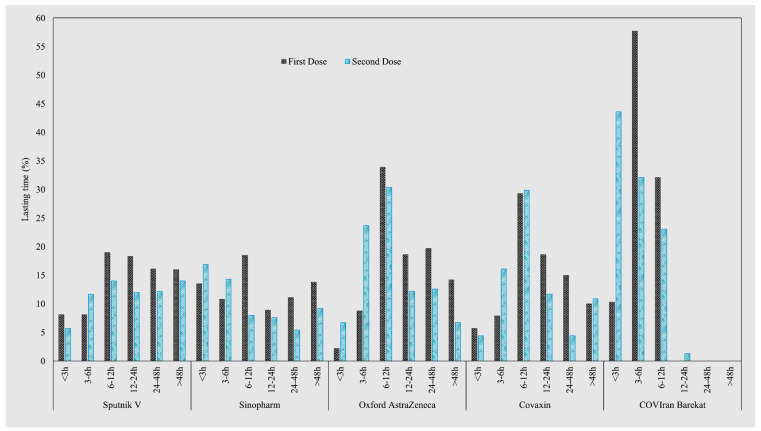
Frequency of Lasting Time for Post-COVID-19 Vaccination Signs and Symptoms by Dose.

Furthermore, the post-vaccination signs and symptoms lasted up to 12 h in roughly 59% of participants, up to 24 h in 14.1%, up to 48 h in 12.6%, and for more than 48 h in 12.4% (Figure 2).

The lasting time of signs and symptoms was significantly highest for the Oxford AstraZeneca vaccine in both the first and second doses, whereas it was significantly lowest for COVIran Barekat after the first dose and Sinopharm after the second dose in comparison with the other vaccines (Table 3).

**Figure 3:**
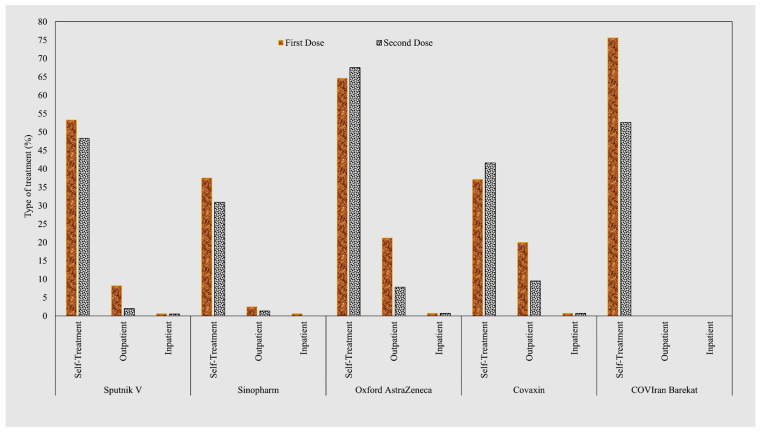
Frequency of Type of Treatment for Post-COVID-19 Vaccination Signs and Symptoms by Dose.

Most of the post-vaccination signs and symptoms were self-limited in all of the vaccination groups. The percentage of vaccinated participants who needed outpatient treatments was highest for the Oxford AstraZeneca and Covaxin vaccines. In the COVIran Barekat group, no one needed to receive outpatient treatment (Figure 3).

### 3.3. Post-COVID-19 Vaccination Local Signs and Symptoms Following the First and Second Doses

Overall, pain and tenderness at the injection site were the most common, and swollen lymph nodes were the least common local signs and symptoms after both the first and second doses of the injected vaccines (Table 4).

After The First Dose: The post-vaccination local signs and symptoms with the significantly highest incidence rate were pain (58.4%), firmness (16.4%), redness (6.2%), and bruising (2.2%) for Oxford AstraZeneca; swelling (16.4%) and petechia (1.4%) for Covaxin; and tenderness (43.6%) and itching (1.3%) for the COVIran Barekat vaccine (Table 4).

After the Second Dose: Similar to the first dose, the post-vaccination local symptoms with the highest incidence rate included pain (48.5%), tenderness (31.5%), firmness (6.3%), swelling (4.4%), and bruising (2.2%) for Oxford AstraZeneca; bruising (2.2%), itching (2.2%), and petechia (0.4%) for Covaxin; and redness (1.6%) for the Sinopharm vaccine (Table 3). A statistically significant level was observed in the incidence rate of pain (*p* < 0.001), tenderness (*p* < 0.001), and firmness (*p* < 0.01) for the Oxford AstraZeneca vaccine (Table 4).

The incidence rates of all of the post-vaccination local symptoms, except for redness, after the second dose was significantly lower than after the first dose (*p* < 0.05) (Table 4).

### 3.4. Post-COVID-19 Vaccination Systemic Signs and Symptoms Following the First and Second Doses

Overall, fever, muscle pain, chills, and headache were the most common post-vaccination systemic symptoms after both the first and second doses of the vaccines (Table 5).

After The First Dose: The significantly highest incidence rates of the post-vaccination systemic symptoms were fever (73.4%), muscle pain (60.0%), joint pain (49.6%), chills (63.5%), malaise (46.7%), headache (45.3%), sweating (12.4%), and palpitation (6.6%) for Oxford AstraZeneca; diarrhoea (27.9%), abdominal pain (21.4%), and nausea and vomiting (22.1%) for Covaxin; and drowsiness (74.4%) and blurred vision (6.4%) for the COVIran Barekat vaccine. Moreover, dyspnoea (2.9%) and chest pain (3.3%) for Oxford AstraZeneca, and pruritus (0.6%) and allergic reactions (0.9%) for the Sinopharm vaccine showed the highest incidence rates; however, they were not significant. Only one case of seizure was reported for Sputnik V (Table 5).

After the Second Dose: The significantly highest incidence rate of the post-vaccination systemic symptoms included fever (43.3%), muscle pain (45.9%), joint pain (34.4%), chills (37.0%), malaise (36.7%), and blurred vision (5.6%) for Oxford AstraZeneca; headache (38.7%), nausea and vomiting (14.6%), abdominal pain (21.4%), diarrhoea (17.5%), and sweating (7.3%) for Covaxin; and drowsiness (69.2%) for the COVIran Barekat vaccine (Table 4). Furthermore, palpitations (4.4%) and allergic reactions (0.7%) for Oxford AstraZeneca; chest pain (2.5%) for Sinopharm; and pruritus (0.3%) and dyspnoea (1.0%) for Sputnik V showed insignificantly higher incidence rates (Table 5). Except for palpitations and shortness of breath, the rate of all other systemic symptoms after the first dose was much lower than after the second dose (Table 5).

## 4. Discussion

In this prospective cohort study, we investigated and compared the incidence rate of the presented signs and symptoms following the administration of the first and second doses of different kinds of COVID-19 vaccines that were in use for healthcare workers.

Though most publications reported the post-vaccine signs and symptoms of Pfizer–BioNTech, Moderna, and Oxford AstraZeneca vaccines [14,29,30,31], the evidence on others is lacking. As such, evidence for Sinopharm, Sputnik V, Covaxin, and especially COVIran Barekat and a comparison between them by dose over 28 days post-vaccine is scarce. To the best of our knowledge, this is the first study assessing the signs and symptoms rates following the administration of different types of COVID-19 vaccines by dose over 28 days.

In this study, about 73% of healthcare workers reported at least one post-vaccine sign or symptom, developing mostly within the first 12 h and lasting up to 12 h. The onset time of signs and symptoms was significantly higher for the COVIran Barekat and Oxford AstraZeneca vaccines in both the first and second doses. In addition, the Oxford AstraZeneca vaccine showed the significantly longest-lasting post-vaccine signs and symptoms. Recent studies, in line with our study, have shown that post-vaccine signs and symptoms appear within the first 12 h [13,24].

Overall, for all injected COVID-19 vaccines, pain and tenderness at the injection site were the most common, and swollen lymph nodes were the least common local signs and symptoms after both the first and second doses of vaccines. We did not observe any serious reactions. After the first dose of COVID-19 vaccines, the most significant rate of local signs and symptoms included pain, firmness, redness, and bruising for Oxford AstraZeneca; swelling and petechia for Covaxin; and tenderness and itching for the COVIran Barekat vaccine. After the second dose, the Oxford AstraZeneca vaccine showed the most significant rate of pain, tenderness, and firmness. In a similar study carried out on 503 healthcare workers in Birjand (Southern Khorasan province, Iran) [12], pain at the injection site was the most common local symptom after receiving the first dose of the Sputnik V, Oxford AstraZeneca, and Covaxin vaccines. In the Covaxin group, it was significantly higher than in the Covaxin group, whereas in our study, swelling and petechial had the significantly highest rate after receiving the Covaxin vaccine. In another study conducted on 672 healthcare workers after taking the first dose of the Oxford AstraZeneca vaccine in Ethiopia, a total of 75.8% reported local symptoms, among which pain (65.4%) and tenderness (57.8%) were the most common post-vaccine symptoms [24]. In addition, in a prospective observational study conducted on 627,383 persons who had received one or two doses of the Pfizer–BioNTech vaccine or one dose of the Oxford AstraZeneca vaccine in the UK, tenderness (49.3%) and local pain (19.1%) around the injection site were the most frequently reported local effects after the first dose of the Oxford AstraZeneca vaccine [22].

Regarding the systemic signs and symptoms, all together, fever, muscle pain, chills, and headache were the most common post-vaccination systemic symptoms after both the first and second doses of all vaccines. These systemic signs and symptoms had the highest significant incidence rate in the Oxford AstraZeneca vaccine. This result is approximately in accordance with those conducted in the UK [22] and Ethiopia [24], in which headache, fatigue, chills, and myalgia were the most prevalent systemic signs and symptoms following injection of the Oxford AstraZeneca vaccine. In addition, in a study by Saeed et al. (2021) in the United Arab [32], fatigue and headache were the most common systemic symptoms post the first and second doses of the Sinopharm vaccine. Moreover, Zare et al. (2021) [20] reported fatigue, muscle pain, and fever as the most common systemic signs and symptoms following the first dose of the Sputnik V, Oxford AstraZeneca, and Covaxin vaccines that are similar to the current study. These post-reactions were significantly highest for Oxford AstraZeneca. Oxford AstraZeneca is an adenoviral vector vaccine that does not replicate and is based on adenoviruses that have been rendered inactive by removing the E1A and E1B gene regions. These recombinant viruses have a sequence encoding spike protein, permitting its production in the infected cell, and the spike antigen cDNA is added to their genome. Then, infected cells show a spike of T cells on their surface. This vaccination induces potent immunological reactions and boosts cellular and humoral protection [33]. It is more likely than not that the Oxford AstraZeneca vaccine, when compared to others, has a greater ability to activate upstream chemical components that cause pain and fever. In terms of the comparison of post-vaccine signs and symptoms between the first and second doses, we found that the rate of all post-COVID-19 vaccine signs and symptoms, apart from palpitation, dyspnoea, and redness, significantly decreased after the second dose. In line with our results, an Iranian study reported a significant reduction in most post-vaccine signs and symptoms in the second dose of the Sputnik V vaccine in comparison with the first dose [34]. In contrast, some recent studies documented a significantly increased rate in most post-vaccine signs and symptoms in the second dose of Sputnik V, Sinopharm, Pfizer-BioNTech, and AstraZeneca vaccines compared to the first dose [13,32].

## 5. Strength and Limitations

In the current study, we compared the wide spectrum of signs and symptoms appearing following the administration of five different kinds of COVID-19 vaccines by dose that were in use in Iran. This is the first study to report signs and symptoms following the administration of the COVIran Barekat vaccine. A high response rate of about 85.5% shows an acceptable quality of our survey and a low risk of sampling bias. Assessing reported signs and symptoms over 28 days post-vaccine provided a great opportunity for us to realize any late post-vaccine reactions that are lacking in publications. On the other hand, some limitations could be considered. Firstly, it is possible that the missing data on serious signs and symptoms following the first dose was higher among those healthcare workers who had refused to participate in the study due to their low motivation, leading to an increased risk of selection bias. Secondly, certain measurements, such as the onset and lasting time of signs and symptoms, might be distorted by the recall bias.

## 6. Conclusions

Pain and tenderness at the injection site and fever, muscle pain, chills, and headache were the most prevalent post-vaccination local and systemic signs and symptoms after both the first and second doses. Most signs and symptoms started within 12 h and lasted for 12 h. The first dose of vaccines developed significantly more prevalent signs and symptoms than the second dose. Both the first and second doses of the Oxford AstraZeneca vaccine showed the highest incidence rate, onset, and lasting time of the post-vaccination signs and symptoms compared to other vaccines. Given that almost all reported post-vaccine signs and symptoms in this comparative study were brief and non-life-threatening, it can boost the public’s confidence in making informed decisions about vaccination. Furthermore, the findings of this independent study may have an impact on reaching a consensus on the safety of COVID-19 vaccines. Nevertheless, more precise gender- and age-based investigations are warranted to find out the contributing factors affecting the safety of different platforms of COVID-19 vaccines.

## Figures and Tables

**Table 1:** Basic Characteristics of the Participants.

Characteristics		N (%)
Gender	Male	651 (43.3)
	Female	849 (56.6)
Occupation	Healthcare professionals	1077(71.8)
	Technicians	267 (17.8)
	Clerks	156 (10.4)
Previous COVID-19	Yes	871 (58.1)
	No	629 (41.9)
Comorbidity	No	1362 (90.8)
	DM	15 (1)
	HTM	25 (1.7)
	HTN and DM	6 (0.4)
	TD	23 (1.5)
	Others	69 (4.6)

N: number of participants; DM: diabetes mellitus; HTN: hypertension; TD: thyroid disorders.

**Table 2:** Comparison of Basic Characteristics of Participants by Vaccine Type.

Vaccine Type	FD N (%)	SD N (%)	Age (Mean-SD)	Gender N (%)	COVID-19 Positive History N (%)	Comorbidity N (%)
Sputnik V	683 (45.53)	667 (45.49%)	35.56 (8.02)	M: 287 (42) F: 396 (58)	350 (51.2)	DM: 8 (1.2) HTN: 9 (1.3) DM+HTN: 2 (0.3) TD: 16 (2.3) Other: 27 (3.9)
Sinopharm	325 (21.66)	314 (21.41)	37.57 (8.98)	M: 152 (46.8) F: 173 (53.2)	210 (64.6)	DM: 4 (1.2) HTN: 8 (2.5) DM+HTN: 3 (0.9) TD: 4 (1.2) Other: 27 (8.3)
Oxford AstraZeneca	274 (16.46)	270 (18.41)	36.59 (7.75)	M: 138 (50.4) F: 136 (49.6)	171 (62.4)	DM: 3 (1.1) HTN: 5 (1.8) DM+HTN: 0 (0) TD: 1 (0.4) Other: 7 (2.55)
Covaxin	140 (0.09)	137 (0.09)	37.08 (6.29)	M: 40 (28.6) F: 100 (71.4)	81 (57.9)	DM: 0 (0) HTN: 1 (0.7) DM+HTN: 0 (0) TD: 2 (1.4) Other: 7 (5)
COVIran Barekat	78 (0.05)	78 (0.05)	36.50 (6.17)	M: 34 (43.6) F: 44 (56.4)	59 (75.6)	DM: 0 (0) HTN: 2 (2.6) DM+HTN: 1 (1.3) TD: 0(0) Other: 1 (1.3)
Total	1500	1466				
Test *p*-Value			*Kruskal–Wallis p*-Value = *0.003*	*χ*^2^*p*-Value = *0.001*	*χ*^2^*p*-Value < *0.001*	*χ*^2^*p*-Value = *0.859*

N: number of participants; FD: first dose; SD: second dose; M: male; F: female; DM: diabetes mellitus; HTN: hypertension; TD: thyroid disorders.

**Table 3:** Pairwise Comparison of Mean Ranks of Onset and Lasting Times for Post-COVID-19 Vaccination Signs and Symptoms by Dose.

Vaccine Name	Onset Time		Lasting Time	
	FD MR	SD MR	FD MR	SD MR
Sinopharm and Covaxin	618.57–629.79	623.28–708.51 *	635.98–750.91	590.76–765.92 **
Sinopharm and Sputnik V	618.57–748.20 **	623.28–681.50 *	635.98–784.13 **	590.76–758.98 **
Sinopharm and Oxford AstraZeneca	618.57–915.52 **	623.28–925.58 **	635.98–880.52 **	590.76–849.78 **
Sinopharm and COVIran Barekat	618.57–957.35 **	623.28–1000.85 **	635.98–475.75 *	590.76–621.72
Covaxin and Sputnik V	629.79–748.20 *	708.51–681.50	750.91–784.13	765.92–758.98
Covaxin and Oxford AstraZeneca	629.79–915.52 **	708.51–925.58 **	750.91–880.52 *	765.92–849.78
Covaxin and COVIran Barekat	629.79–957.35 **	708.51–1000.85 **	750.91–475.75 **	765.92–621.72
Sputnik V and Oxford AstraZeneca	748.20–915.52 **	681.50–925.58 **	784.13–880.52 *	758.98–849.78 *
Sputnik V and COVIran Barekat	748.20–957.35 **	681.50–1000.85 **	784.13–475.75 **	758.98–621.72 *
Oxford AstraZeneca and COVIran Barekat	915.52–957.35	925.58–1000.85	880.52–475.75 **	849.78–621.72 **

MR: mean rank; FD: first dose; SD: second dose; * Kruskal–Wallis *p*-value < 0.05; ** Kruskal–Wallis *p*-value < 0.001.

**Table 4:** Frequencies of Post-COVID-19 Vaccination Local Signs and Symptoms by Dose.

Local Signs and Symptoms																				
Vaccine Name	N	N	Pain N (%)		Tenderness N (%)		Redness N (%)		Bruising N (%)		Swelling N (%)		Petechia N (%)		Itching N (%)		Firmness N (%)		Swollen Lymph Nodes N (%)	
	FD	SD	FD	SD	FD	SD	FD	SD	FD	SD	FD	SD	FD	SD	FD	SD	FD	SD	FD	SD
Sputnik V	683	667	305 (44.7)	219 (32.8)	143 (20.9)	59 (8.8)	19 (2.8)	3 (0.4)	3 (0.4)	8 (1.2)	27 (4)	11 (1.6)	4 (0.6)	2 (0.3)	3 (0.4)	3 (0.4)	44 (6.4)	18 (2.7)	4 (0.6)	3 (0.4)
Sinopharm	325	314	127 (39.1)	83 (26.4)	53 (16.3)	26 (8.3)	2 (0.6)	5 (1.6)	1 (0.3)	0 (0.0)	6 (1.8)	4 (1.3)	0 (0)	0 (0)	3 (0.9)	0 (0)	11 (3.4)	4 (1.3)	0 (0.0)	0 (0.0)
Oxford AstraZeneca	274	270	160 (58.4)	131 (48.5)	112 (40.9)	85 (31.5)	17 (6.2)	4 (1.5)	6 (2.2)	6 (2.2)	31 (11.3)	12 (4.4)	0 (0.0)	0 (0.0)	0 (0.0)	2 (0.7)	45 (16.4)	17 (6.3)	0 (0.0)	0 (0.0)
Covaxin	140	137	62 (44.3)	36 (26.3)	44 (31.4)	16 (11.7)	2 (1.4)	1 (0.7)	1 (0.7)	3 (2.2)	23 (16.4)	3 (2.2)	2 (1.4)	1 (0.7)	1 (0.7)	3 (2.2)	21 (15)	7 (5.1)	1 (0.7)	1 (0.7)
COVIran Barekat	78	78	12 (15.4)	5 (6.4)	34 (43.6)	14 (17.9)	2 (2.6)	0 (0)	0 (0)	0 (0)	5 (6.4)	3 (3.8)	0 (0)	0 (0)	1 (1.3)	0 (0)	2 (2.6)	1 (1.3)	0 (0.0)	0 (0.0)
Total	1500	1466	666 (44.4)	474 (31.6)	386 (25.7)	200 (13.6)	42 (2.8)	13 (0.9)	11 (0.7)	17 (1.2)	92 (6.1)	33 (2.3)	6 (0.4)	3 (0.2)	8 (0.5)	8 (0.5)	123 (8.2)	47 (3.2)	5 (0.3)	4 (0.3)
*χ*^2^*p*-Value			<0.001	<0.001	<0.001	<0.001	0.001	0.282	0.037	0.074	<0.001	0.055	0.134	0.477	0.489	0.055	<0.001	0.004	0.382	0.458

N: number of participants; FD: first dose; SD: second dose.

**Table 5:** Frequencies of Post-COVID-19 Vaccination Systemic Signs and Symptoms by Dose.

Systemic Signs and Symptoms																				
Vaccine Type	N	N	Fever N (%)		Muscle Pain N (%)		Join Pain N (%)		Chills N (%)		Malaise N (%)		Dyspnoea N (%)		Headache N (%)		Blurred Vision N (%)		Nausea and Vomiting N (%)	
	FD	SD	FD	SD	FD	SD	FD	SD	FD	SD	FD	SD	FD	SD	FD	SD	FD	SD	FD	SD
Sputnik V	683	667	310 (45.4)	220 (33)	288 (42.2)	193 (28.9)	160 (23.4)	117 (17.5)	237 (34.7)	158 (23.7)	229 (33.5)	126 (18.9)	12 (1.8)	7 (1.0)	193 (28.3)	124 (18.6)	11 (1.6)	6 (0.9)	37 (5.4)	20 (3)
Sinopharm	325	314	79 (24.3)	51 (16.2)	52 (16)	33 (10.5)	17 (5.2)	8 (2.5)	40 (12.3)	31 (9.9)	57 (17.5)	43 (13.7)	6 (1.8)	2 (0.6)	78 (24)	61 (19.4)	2 (0.6)	7 (2.2)	10 (3.1)	9 (2.9)
Oxford AstraZeneca	274	270	201 (73.4)	118 (43.7)	178 (65)	124 (45.9)	136 (49.6)	93 (34.4)	174 (63.5)	100 (37)	128 (46.7)	99 (36.7)	8 (2.9)	0 (0.0)	124 (45.3)	82 (30.4)	8 (2.9)	15 (5.6)	12 (4.4)	10 (3.7)
Covaxin	140	137	54 (38.6)	16 (11.7)	43 (30.7)	20 (14.6)	15 (10.7)	9 (6.6)	40 (28.6)	11 (8)	24 (17.1)	24 (17.5)	1 (0.7)	0 (0.0)	59 (42.1)	53 (38.7)	2 (1.4)	1 (0.7)	31 (22.1)	20 (14.6)
COVIran Barekat	78	78	47 (60.3)	23 (29.5)	24 (30.8)	12 (15.4)	3 (3.8)	6 (7.7)	29 (37.2)	14 (17.9)	17 (21.8)	13 (16.7)	0 (0)	0 (0.0)	21 (26.9)	9 (11.5)	5 (6.4)	1 (1.3)	0 (0)	0 (0)
Total	1500	1466	691 (46.1)	428 (29.2)	585 (39)	382 (26.1)	331 (22.1)	233 (15.9)	520 (34.7)	314 (21.4)	455 (30.3)	305 (20.8)	27 (1.8)	9 (0.6)	475 (31.7)	329 (22.5)	28 (1.9)	30 (2)	90 (6)	59 (4)
*χ*^2^ *p*-Value			<0.001	<0.001	<0.001	<0.001	<0.001	<0.001	<0.001	<0.001	<0.001	<0.001	0.365	0.280	<0.001	<0.001	0.009	<0.001	<0.001	<0.001
**Vaccine Type**	**N**	**N**	**Diarrhoea** **N (%)**		**Chest Pain N (%)**		**Abdominal Pain** **N (%)**		**Seizure** **N (%)**		**Drowsiness** **N (%)**		**Sweating** **N (%)**		**Pruritus** **N (%)**		**Palpitation** **N (%)**		**Allergic Reactions** **N (%)**	
	**FD**	**SD**	**FD**	**SD**	**FD**	**SD**	**FD**	**SD**	**FD**	**SD**	**FD**	**SD**	**FD**	**SD**	**FD**	**SD**	**FD**	**SD**	**FD**	**SD**
Sputnik V	683	667	8 (1.2)	5 (0.7)	13 (1.9)	13 (1.9)	9 (1.3)	8 (1.2)	1 (0.1)	0 (0)	84 (12.3)	50 (7.5)	54 (7.9)	20 (3)	2 (0.3)	2 (0.3)	26 (3.8)	28 (4.2)	1 (0.1)	0 (0)
Sinopharm	325	314	9 (2.8)	10 (3.2)	6 (1.8)	8 (2.5)	3 (0.9)	3 (1)	0 (0)	0 (0)	33 (10.2)	35 (11.1)	4 (1.2)	7 (2.2)	2 (0.6)	0 (0)	8 (2.5)	12 (3.8)	3 (0.9)	2 (0.6)
Oxford AstraZeneca	274	270	5 (1.8)	14 (5.2)	9 (3.3)	6 (2.2)	0 (0)	2 (0.7)	0 (0)	0 (0)	57 (20.8)	64 (23.7)	34 (12.4)	18 (6.7)	0 (0)	0 (0)	18 (6.6)	12 (4.4)	0 (0)	2 (0.7)
Covaxin	140	137	39 (27.9)	24 (17.5)	0 (0)	2 (1.5)	30 (21.4)	17 (12.4)	0 (0)	0 (0)	12 (8.6)	11 (8)	7 (5)	10 (7.3)	0 (0)	0 (0)	3 (2.1)	2 (1.5)	0 (0)	0 (0)
COVIran Barekat	78	78	0 (0)	0 (0)	0 (0)	0 (0)	0 (0)	0 (0)	0 (0)	0 (0)	58 (74.4)	54 (69.2)	2 (2.6)	2 (2.6)	0 (0)	0 (0)	0 (0)	2 (2.6)	0 (0)	0 (0)
Total	1500	1466	61 (4.1)	53 (3.6)	28 (1.9)	29 (2)	42 (2.8)	30 (2)	1 (0.1)	0 (0)	244 (16.3)	214 (14.6)	101 (6.7)	57 (3.9)	4 (0.3)	2 (0.1)	55 (3.7)	56 (3.8)	4 (0.3)	4 (0.3)
*χ*^2^*p*-Value			<0.001	<0.001	0.128	0.667	<0.001	<0.001	0.879		<0.001	<0.001	<0.001	0.008	0.589	0.663	0.019	0.565	0.138	0.191

N: number of participants; FD: first dose; SD: second dose.

## Data Availability

Datasets that are minimally required to replicate the outcomes of the study will be made available upon reasonable request. In this case, the corresponding author will be responsible for providing the requested data.

## Abbreviations

COVID-19: coronavirus disease 2019; SARS-CoV-2: severe acute respiratory syndrome coronavirus 2; WHO: World Health Organization; mRNA: messenger ribonucleic acid; FDA: Food and Drugs Administration, EMA: European Medicines Agency; SD: standard deviation; ANOVA: analysis of variance; USA: United States of America; UK: United Kingdom.
